# Arthroscopic thermal capsular shrinkage: a successful treatment option for patients with chronic lateral ankle instability?

**DOI:** 10.1186/1753-6561-6-S4-O32

**Published:** 2012-07-09

**Authors:** J White, A Zubairy, M Hakimi

**Affiliations:** 1 University of Manchester, UK; 2 East Lancashire Hospitals Trust, UK

## Introduction

Thermal capsular shrinkage is a well recognised procedure in the management of shoulder instability, but little data has been collected with regards to other joints. This surgical technique offers an alternative treatment to ligament reconstruction in chronic lateral ankle instability.

## Objectives

This study looks at the outcomes of thermal capsular shrinkage in patients with chronic lateral ankle instability. It aims to audit the results of thermal capsular shrinkage against published literature, and to devise a recommended rehabilitation protocol.

## Methods

Data was collected from 26 patients (12 males, 12 right ankles, mean age 32.88 years) from November 2004 to March 2011. 20 patients were available for follow up. Preoperative MRI scans and stress radiographs were taken. The patients were all assessed using the American Orthopaedic Foot and Ankle Society (AOFAS) ankle-hindfoot scale, Manchester-Oxford Foot Questionnaire, EuroQol and the Visual Analogue Scale Foot and Ankle. Reviews were held at an average of 27.4 months postoperatively.

## Results

Postoperative AOFAS scores improved by a mean of 40 points (P<0.05). 12 patients rated their satisfaction as excellent or very good, 7 as good and 1 as poor. No revision procedures were required. Physiotherapy regimes were analysed, and then collaborated to form what we believe to be the optimum postoperative management for these patients.

## Conclusions

Results show that this minimally invasive arthroscopic surgical technique is a successful treatment option with minimal morbidity for patients with chronic lateral ankle instability. No revision procedures were performed.

